# Speech and Language Markers as Longitudinal Predictors of Youth Mental Health: A Systematic Review

**DOI:** 10.1111/eip.70102

**Published:** 2025-10-05

**Authors:** Martin Sellier Silva, Jessica Ahrens, Fiona Meister, Lena Palaniyappan

**Affiliations:** ^1^ Douglas Mental Health University Institute, Department of Psychiatry McGill University Montreal Quebec Canada; ^2^ Integrated Program in Neuroscience McGill University Montreal Quebec Canada; ^3^ Department of Psychiatry Schulich School of Medicine and Dentistry, Western University London Ontario Canada

**Keywords:** communication, prediction, psychopathology, speech, youth mental health

## Abstract

**Introduction:**

Severe mental disorders in young people (< 25 years) are often preceded by subtle changes in communication and thinking, detectable in speech. Speech and language markers are promising for early detection; however, no systematic review has evaluated their prospective utility in predicting mental disorders in youth. We comprehensively reviewed longitudinal studies assessing speech/language markers as predictors of major mental disorder onset or symptom progression in youth.

**Methods:**

We searched for longitudinal studies using recorded speech samples from youth or family members to predict diagnostic changes or symptom severity in major depressive disorder (MDD), psychosis, ADHD, substance use disorder, bipolar disorder, OCD and eating disorders. Risk of bias was assessed using the Newcastle–Ottawa Scale. Our protocol was pre‐registered (CRD42024579798).

**Results:**

Of 2260 articles, 11 studies met inclusion criteria, covering MDD (*n* = 3), psychosis (*n* = 5) and ADHD (*n* = 3). No eligible studies were found for OCD, substance use, bipolar or eating disorders. Both manual and computational speech analyses were used, with speech samples from parents and youth. Predictive speech/language markers included parental expressed emotion (MDD, ADHD), formal thought disorder (psychosis) and acoustic/linguistic features (psychosis, ADHD). Study quality was moderate to good (mean score: 5.45/8).

**Conclusions:**

Externally validated longitudinal studies on the predictive value of speech/language markers of youth‐onset mental disorders are scarce, restricted to a few target disorders and do not allow for variations due to the developmental stage of the samples. Nonetheless, existing studies highlight the potential of applying Natural Language Processing methods to speech samples from both youth and parents for early identification.

## 1 Introduction

Mental disorders are highly prevalent in youth, with Global Burden of Disease indicating one of 10 individuals between 5‐ and 24‐year‐old living with a diagnosable mental disorder in 2019 (Kieling et al. 2024). Furthermore, mental disorders in this age group account for approximately 20% of the overall burden of disease (Kieling et al. 2024). To reduce this enormous burden, focusing on prevention is imperative. Mental health prevention and promotion initiatives have shown promise in reducing or delaying the onset of mental disorders (Arango et al. 2018; Singh et al. 2022); however, a major barrier for implementing these approaches is our inability to reliably forecast the onset of mental disorders.

For targeted interventions aimed at prevention, more accurate methods to predict disorders' onset are needed. Specifically, for preventive interventions to be implemented, we need to know *who* will develop *which* condition and *when*. To this end, several candidate markers and tools have been tested, including genetic risk (Gatt et al. 2015), magnetic resonance imaging markers (Lukow et al. 2021) and environmental risk factors (Schmitt et al. 2014). While considerable research has focused on identifying biomarkers to help clinicians diagnose or predict mental disorders' onset, these remain insufficiently accurate for clinical implementation or inaccessible due to resource constraints (Abi‐Dargham et al. 2023). Psychopathological antecedents are particularly valuable as they can be obtained without invasive procedures and often relate directly to mental disorders' constructs (e.g., poor sleep and depression, subthreshold psychosis and schizophrenia; Uher et al. 2024). Most prospective studies of these antecedents have relied on self‐reports, which require awareness, memory and willingness to report—all of which are subject to bias, affecting generalisability.

A behavioural antecedent that circumvents self‐report biases is verbal behaviour. The physical and informational properties of our verbalisations (i.e., speech and language) are fundamental in the assessment of mental disorders. For example, reduced speech (alogia) and impaired structure (formal thought disorder; FTD) are core features of schizophrenia (Andreasen 1979), while pressured speech is a diagnostic criterion for bipolar disorder (BD) in the *Diagnostic and Statistical Manual of Mental Disorders* (DSM‐5; American Psychiatric Association 2013). However, generating objective readout of spontaneous speech to aid clinical practice is a more recent approach. Several studies have attempted to classify and diagnose mental health conditions using objective speech variables (Zhang et al. 2022), including schizophrenia (Zaher et al. 2024), BDs (Harvey et al. 2022) and major depressive disorder (MDD) (Koops et al. 2023). The performance of speech/language features in identifying mental disorders cross‐sectionally varies widely, ranging from chance levels to high accuracy, with some studies reporting F1 scores as high as 0.95 (Afshan et al. 2018). This suggests the potential of speech/language as a high‐yield marker of psychopathology. However, the effectiveness of specific features may depend on factors such as demographic characteristics, the type of disorder and even sample size, all of which must be carefully considered (Low et al. 2020).

Using speech/language markers to detect or predict mental disorders offers various advantages. First, speech is an easily accessible and non‐invasive by‐product of human interaction. The provision of human interaction remains the *raison d'être* of clinical practice in mental health. Second, speech allows for remote monitoring. Given that underserved populations struggle to access mental health resources (Bartram 2019), digital tools leveraging speech offer a more inclusive, cost‐effective and accessible assessment medium. Moreover, speech features associated with mental disorders are increasingly defined using computational methods, such as Natural Language Processing (NLP), which are highly scalable, adding incremental value to clinical assessments (Mackinley et al. 2021; Silva et al. 2021, 2023).

Despite the growing body of literature on mental health and speech/language, most studies follow a cross‐sectional design (Low et al. 2020), primarily differentiating already diagnosed clinical samples from healthy populations—thus not testing *predictive utility*. To assess the utility of speech/language markers in predicting mental disorders before their onset, the ideal study design would assess speech in undiagnosed populations, with a prospective follow‐up to diagnose new onset mental disorders. To the best of our knowledge, no systematic literature review has been conducted to locate and appraise such prospective studies. A prior attempt did not focus on youth or prospective prediction (Dikaios et al. 2023). We aim to fill this gap by providing a comprehensive evaluation of the literature on speech/language as a prospective marker of mental disorder development in youth.

## 2 Methods

We registered the protocol for this study with the International Prospective Register of Systematic Reviews website (CRD42024579798).

### 2.1 Search Strategy and Selection

We conducted a systematic review of the literature to identify studies that longitudinally examined speech/language markers as a predictor of the following major mental disorders where speech is known to be affected: MDD (Koops et al. 2023), psychosis (Zaher et al. 2024), attention deficit hyperactivity disorder/attention deficit disorder (ADHD/ADD; Li et al. 2024), substance use disorder (SUD; Agurto et al. 2025), BD (Briganti and Lechien 2025) and obsessive compulsive disorder (OCD; Cassol et al. 2010). We also included eating disorders (ED) to accommodate multiple, albeit non‐specific linguistic changes reported in several studies (Burke 1991; Cuteri et al. 2022; Maćkowska et al. 2022; Spinczyk et al. 2018). These disorders often emerge during youth and frequently persist or recur in adulthood (Girela‐Serrano et al. 2024).

Articles were retrieved from PubMed (1951—February 15, 2025), Ovid (1947—February 15, 2025) and Google Scholar (1960—February 15, 2025). Detailed information on keywords and the search strategy used for each database is provided in the Appendix A.

We conducted three stages of review: screening titles, abstracts and full texts. For each stage, MSS and JA independently screened the articles and resolved conflicts together on Covidence (Veritas Health Innovation 2016), which removed (*n* = 91) duplicates. Articles were included if they met the a priori criteria: (1) longitudinal design; (2) use of recorded speech samples from either youths themselves or a family member; (3) included participants between 0 and 25 years old at baseline; (4) aimed to prospectively predict changes in mental health diagnosis or symptom severity; and (5) used a validated questionnaire or diagnostic interview to examine disorder outcomes. We excluded reviews, meta‐analyses, case studies/reports, validation studies or papers which solely examined verbal ability, cognitive tests or developmental speech impairments (e.g., stuttering, reading deficits). We also excluded papers written in any other language than French, English or Spanish.

Data extraction was conducted using Covidence (Veritas Health Innovation 2016). MSS and JA independently went through each of the studies extracting the pre‐determined relevant data. Extracted data included: title and publication year of the article, aim of the study, length of follow‐up(s), statistical methods, linguistic measures, inclusion and exclusion criteria, number of participants, mean age, sex, ethnicity, speech marker used, statistical methods and results and prediction results. Once again, conflicts were resolved by re‐examining the articles together.

A risk of bias assessment was completed using an adaptation of the Newcastle–Ottawa Scale (Wells et al. 2009) in which JA and MSS independently rated the studies, resolving conflicts through re‐examining. Specifically, we adapted the scale for sufficient follow‐up duration, which was determined by looking at the peak age of onset of the mental disorders based on an extensive recent synthesis (Solmi et al. 2022). We then determined whether the mean age at follow‐up in the included sample was close enough (1 year below or older) to this peak age when deriving bias scores on adequacy of follow‐up.

## 3 Results

### 3.1 Study Characteristics

**FIGURE 1:**
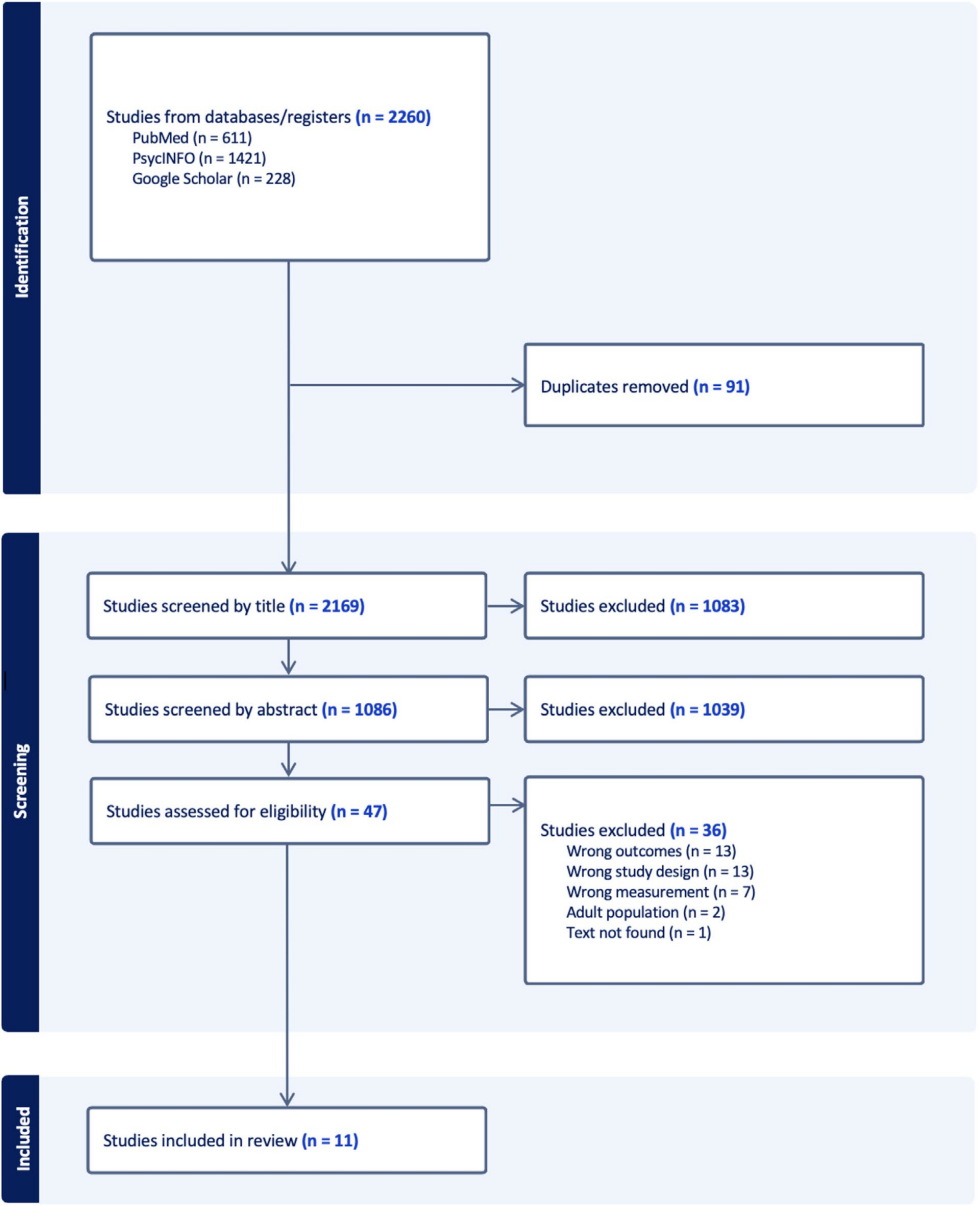
PRISMA flowchart.

The results of our searches are included in Figure 1. Of the initial 2260 articles, 11 studies were included in the review. The studies had an average risk of bias score of 5.45 stars (SD = 1.81) out of a possible 8, indicating moderate to good quality (see Table 1).

**TABLE 1:** Risk of bias assessment using the Newcastle–Ottawa Scale.

Author (Year)	Exposed cohort representativeness	Non‐exposed cohort selection	Exposure ascertainment	Outcome not present at start	Cohort comparability	Outcome assessment	Sufficient follow‐up duration	Follow‐up adequacy	Total
Agurto et al. (2020)	1	0	1	1	1	1	1	1	7
Allely et al. (2013)	0	1	1	1	1	0	0	0	4
Asarnow et al. (1993)	0	0	1	0	1	1	1	1	5
Bearden et al. (2011)	1	1	1	1	1	1	0	1	7
Bedi et al. (2015)	1	1	1	1	1	1	1	1	8
Burkhouse et al. (2012)	1	1	1	1	1	1	0	1	7
Corcoran et al. (2018)	1	1	1	1	1	1	1	0	7
Gooding et al. (2013)	0	0	1	1	1	0	1	0	4
Ooi et al. (2013)	1	1	1	1	1	0	0	0	5
Peris and Baker 2000	0	0	0	0	1	1	1	0	3
Pauli‐Pott et al. (2021)	0	0	0	0	1	1	0	1	3

*Note:* A score of 1 indicates the criterion was met; 0 indicates it was not. A total score of 8 represents the lowest risk of bias, while 0 would be the highest risk.

Half of the included studies had a control group for comparison with participants at risk of developing a mental disorder (Allely et al. 2013; Bearden et al. 2011; Burkhouse et al. 2012; Corcoran et al. 2018; Gooding et al. 2013; Ooi et al. 2013), while the other half did not (Agurto et al. 2020; Asarnow et al. 1993; Bedi et al. 2015; Pauli‐Pott et al. 2021; Peris and Baker 2000) (Table 2). Average participant age at baseline ranged from 4.5 years old (Peris and Baker 2000) to 21.78 years old (Bearden et al. 2011) (Table 2). Among studies reporting ethnicity (Agurto et al. 2020; Asarnow et al. 1993; Bearden et al. 2011; Bedi et al. 2015; Burkhouse et al. 2012; Corcoran et al. 2018; Peris and Baker 2000), most participants were Caucasian (Mean = 60%) (Table 2). Sex varied depending on the outcome of interest; studies on MDD had an equal amount of female and male participants (mean percentage of male = 45%), while males were generally predominant in psychosis (mean percentage of male = 66%) and ADHD studies (mean percentage of male = 62%) (Table 2). All included studies recorded speech in English language excepted for one (Pauli‐Pott et al. 2021).

**TABLE 2:** Characteristics of studies included in the review.

Author (Year)	Control	Patients (*n*)	Controls (*n*)	Patient age (M ± SD)	Control age (M ± SD)	Patients sex (M/F)	Controls sex (M/F)	Patient ethnicity (% Caucasian)	Control ethnicity (% Caucasian)	Follow‐up duration	Speech measure
ADHD studies (*n* = 3)											
Allely et al. (2013)	Yes	58	111	1^a^	NA	40/18	76/35	NA	NA	6 years	Vocalisation rate/frequency
Pauli‐Pott et al. (2021)	No	138	NA	4.85 (0.51)	NA	85/53	NA	NA	NA	3 years	EE‐criticism EE‐positive relationship
Peris and Baker (2000)	No	69	NA	4.5 (0.6)	NA	45:46	NA	72%	NA	4 years	EE‐criticism, EE‐emotional overinvolvement
Psychosis studies (*n* = 5)											
Agurto et al. (2020)	No	32	NA	21.78 (3.59)	NA	21/11	NA	53%	NA	3–16 months	Pitch variation, spectral characterisation, vowel space, voice quality, changes in rhythm vocalisation rate/frequency
Bearden et al. (2011)	Yes	54	51	17.10 (3.78)	16.2 (2.7)	38/16	31/20	59%	49%	2 years (every 6 months)	Loose association, illogical thinking, poverty of content
Bedi et al. (2015)	No	34	NA	21.35 (3.59)	NA	23/11	NA	38%	NA	2.5 years (every 3 months)	Semantic coherence, parts of speech. Phrase length
Corcoran et al. (2018)	Yes	93	21	18.39 (4.06)	18.0 (2.8)	62/31	13/8	48%	67%	2–2.5 years	Semantic coherence, parts of speech. Phrase length
Gooding et al. (2013)	Yes	74	130	NA	NA	NA	NA	NA	NA	8.23 years (average)	Poverty of content, poverty of speech
MDD studies (*n* = 3)											
Asarnow et al. (1993)	No	26	NA	11.28 (NA)	NA	17:9	NA	92%	NA	1 year	EE‐positive relationship
Burkhouse et al. (2012)^a^	Yes	48	52	9.97 (1.32)	NA	41:59	NA	82%	NA	20 months	EE‐criticism
Ooi et al. (2013)	Yes	15	15	12–13	12–13	9:6	9:6	NA	NA	2 years	Glottal, prosodic, spectral, Teager's Energy Operator

Abbreviation: NA = Not available/not applicable.

^a^ Exact values not available.

Three studies aimed to predict MDD diagnosis (Asarnow et al. 1993; Burkhouse et al. 2012; Ooi et al. 2013), five psychosis (Agurto et al. 2020; Bearden et al. 2011; Bedi et al. 2015; Corcoran et al. 2018; Gooding et al. 2013) and three ADHD (Allely et al. 2013; Pauli‐Pott et al. 2021; Peris and Baker 2000). Despite including other disorders in our search, no studies met our inclusion criteria for predicting OCD, SUD, EDs or BD. Only one study predicted the worsening of depression over time (Asarnow et al. 1993), while the other ones predicted the onset of their respective disorder of interest.

### 3.2 Speech/Language Markers

The included studies used a range of speech/language markers. Four studies investigated speech‐based ratings of parental expressed emotion (EE) in predicting the onset of ADHD (Pauli‐Pott et al. 2021; Peris and Baker 2000) and depression (Asarnow et al. 1993; Burkhouse et al. 2012). Two studies analysed speech‐based ratings of FTD to predict psychosis (Bearden et al. 2011; Gooding et al. 2013). Five studies used objective speech/language markers to predict ADHD (Allely et al. 2013), psychosis (Agurto et al. 2020; Bedi et al. 2015; Corcoran et al. 2018) and depression (Ooi et al. 2013).

Importantly, EE ratings were derived from speech samples obtained from the parents of the children who were assessed for psychopathology. Thus, four studies in total looked at parental speech (Asarnow et al. 1993; Burkhouse et al. 2012; Pauli‐Pott et al. 2021; Peris and Baker 2000) while seven others looked at the youth's own speech. EE was measured using the Five‐Minute Speech Sample (Gottschalk and Glesser 1969), where individuals describe their relationship with family members and the scoring criteria are based on the interviewee's view of their relative (Magaña et al. 1986). Several categories exist within the EE construct, including Emotional Over‐Involvement, Criticism and Hostility, all of which are scored based on frequency. In contrast, FTD is derived from ratings of speech recorded with the Scale for Thought, Language and Communication (Andreasen 1979), categorising symptoms into: negative thought disorder (such as poverty of content and speech) and positive thought disorder (such as tangentiality and derailment). Objective speech markers varied across the studies but in the majority consisted of acoustic measures of participants' speech. Vocalisation rates, both in mothers and children (Allely et al. 2013), are calculated by obtaining and adding up each discrete vocalisation and dividing it by the audio duration. Other acoustic measures used in this review included pitch variation, spectral characterisation, vowel space, voice quality and changes in rhythm (Agurto et al. 2020) as well as Teager Energy Operator (TEO; energy‐based signal periodicity measure) and glottal, prosodic and spectral features (Ooi et al. 2013). Two studies extracted objective linguistic features with NLP such as semantic coherence, phrase length and parts of speech tagging (Bedi et al. 2015; Corcoran et al. 2018).

### 3.3 Main Results

Two studies on MDD found markers of parental EE expressed in speech as predictive of depression severity (Asarnow et al. 1993; Burkhouse et al. 2012); however, they differed in variable selection. Asarnow et al. (1993) defined high EE as an elevated score in either criticism or emotional involvement and used it to predict worsening depression in hospitalised adolescents over a year. Burkhouse et al. (2012) tested only the criticism dimension of EE (EE‐crit) as a predictor of depression onset in high‐risk children over 6 months. The third study (Ooi et al. 2013) looked at several acoustic features (including glottal, prosodic, spectral and TEO‐based features) to predict the onset of MDD in adolescents during a 2‐year‐long follow‐up period. When using a GMM for each of these features, glottal and prosodic features showed higher accuracy of 69.35% and 63.10%, respectively, compared to lower accuracies for TEO (52.08%) and spectral‐based features (53.87%). However, the multichannel approach combining all four features yielded an accuracy of up to 72.77%.

All five studies investigating psychosis prediction identified baseline speech differences between individuals who later developed psychosis and those who remained healthy (Agurto et al. 2020; Bearden et al. 2011; Bedi et al. 2015; Corcoran et al. 2018; Gooding et al. 2013); however, they varied in linguistic markers, statistical methods and follow‐up duration. Gooding et al. (2013) found that negative thought disorder (poverty of speech and poverty of content) significantly predicted schizophrenia‐related psychosis up to 8 years in advance. Bearden et al. (2011) found that poverty of content and referential cohesion were not significant predictors of psychosis onset (1‐year follow‐up; logistic regression) but were higher in converters at follow‐up (MANOVA). Illogical thinking was found to predict psychosis onset with 69% accuracy in a logistic regression model. Agurto et al. (2020) examined acoustic and prosodic speech markers to predict psychosis onset in youth at high risk, finding predictive accuracy (85%–95%) in gender‐adjusted models. Bedi et al. (2015) found that a convex hull classifier—using semantic coherence, phrase length and frequency of determiners—predicted psychosis with 100% accuracy (Area Under the Curve [AUC] = 1.00) over 2.5 years. Corcoran et al. (2018) replicated these findings in a larger sample, with AUCs of 0.87 (internal cohort) and 0.72 (external cohort).

Parental EE to predict ADHD onset was used in two studies (Pauli‐Pott et al. 2021; Peris and Baker 2000). Peris and Baker (2000) found maternal criticism, but not emotional over‐involvement, predicted ADHD diagnosis at approximately 9 years old. Pauli‐Pott et al. (2021) linked parental positive regard to later ADHD, though EE variables were not significant in logistic regression models. Allely et al. (2013) examined parent–infant vocalisation at 12 months but found no predictive relationship with ADHD diagnosis at age 7. A summary of speech variables and results from the included studies can be found in Tables 3 and 4.

**TABLE 3:** Summary of findings from included studies.

Author (year)	Location	Bias risk	Variable	Results	Rating
ADHD studies (*n* = 3)					
Peris and Baker (2000)	Los Angeles, USA	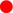	EE‐C, EE‐EOI	EE‐C but not EE‐EOI predicted ADHD diagnosis	Manual
Allely et al. (2013)	Avon, UK	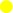	VR/F	VR/F did not predict later ADHD diagnosis	Manual
Pauli‐Pott et al. (2021)	Marburg, Germany	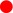	EE‐C, EE‐PR	EE‐C and EE‐PR were significantly predictive of ADHD, but not in logistic regression	Manual
Psychosis studies (*n* = 5)					
Bearden et al. (2011)	Los Angeles, USA	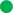	LA, IT, POC	Only IT was a predictor of psychosis onset with logistic regression	Manual
Gooding et al. (2013)	New York, USA	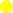	POC, POVS	Higher POC and POVS predicted schizophrenia‐related psychosis onset.	Manual
Agurto et al. (2020)	New York City, USA	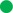	PV, SPC, VS, VQ, CR, VR/F	PV, VS and SPC were the most predictive of conversion to psychosis	Computational
Bedi et al. (2015)	New York City, USA	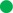	SC, POS, PL	SC, POS and PL predicted conversion to psychosis	Computational
Corcoran et al. (2018)	New York City & Los Angeles, USA	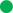	SC, POS, PL	SC, POS and PL predicted conversion to psychosis even in an external sample	Computational
MDD studies (*n* = 3)					
Asarnow et al. (1993)	Los Angeles, USA	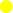	EE‐PR	Higher PR predicted worsening of MDD.	Manual
Burkhouse et al. (2012)	Binghamton, USA	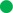	EE‐C	Higher EE‐C predicted MDD onset.	Manual
Ooi et al. (2013)	Melbourne, Australia	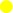	SPC, G, TEO, P	G and P able to predict MDD onset. A combination of G, P, SPC and TEO is an even better predictor	Computational

Bias risk	Speech variables		
	Semantic	Acoustic	Paralinguistic and emotional markers
Low risk 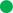 Medium risk 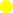 High risk 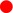	LA—loose association IT—illogical thinking POC—poverty of content POVS—poverty of speech SC—semantic coherence RC—referential cohesion	PV—pitch variation SPC—spectral characterisation VS—vowel space VQ—voice quality CR—changes in rhythm VF/R—vocalisation rate/frequency G—glottal features TEO—Teager energy operator P—prosodic features	EE‐C—EE‐criticism EE‐EOI—EE‐emotional overinvolvement EE‐P—EE‐positive relationship EE‐O—EE‐overall POS—parts of speech PL—phrase length

*Note:* Variables are coded using letters and grouped by speech domain. Bias risk icons reflect quality ratings based on the Newcastle–Ottawa Scale. Low risk indicates a score of 6 or higher, medium risk a score of less than 6 but higher or equal to 4 and high risk corresponds to a score of 3 or less.

**TABLE 4:** Summary of speech marker predictiveness across diagnoses.

Speech domain	ADHD	Psychosis	MDD
Acoustic	Not predictive (0/1)	Predictive (3/6)	Predictive (4/4)
Semantic	Not studied	Predictive (4/5)	Not studied
Paralinguistic and emotional markers	Predictive (2/3)	Predictive (2/2)	Predictive (2/2)

*Note:* The fraction in parenthesis indicates the number of variables out of all the variables analysed in the included studies that were predictive in at least one study.

### 3.4 Statistical Methods

Logistic regression was the most common statistical method used in 8 out of 11 studies (Agurto et al. 2020; Allely et al. 2013; Asarnow et al. 1993; Bearden et al. 2011; Burkhouse et al. 2012; Bedi et al. 2015; Corcoran et al. 2018; Pauli‐Pott et al. 2021). More complex machine learning models, such as Support Vector Machines, Convex Hull classifiers and Gaussian Mixture Models (GMMs), were used in four studies (Agurto et al. 2020; Bedi et al. 2015; Corcoran et al. 2018; Ooi et al. 2013).

## 4 Discussion

To our knowledge, this is the first systematic review examining the role of speech as a longitudinal marker of mental health status change in youth. We aimed to examine the current literature on this topic and identify future research directions. From an initial pool of 2260 studies, 11 met the eligibility criteria. Although we searched for seven different disorders, only studies on MDD, ADHD and psychosis were identified.

Both studies on MDD (Asarnow et al. 1993; Burkhouse et al. 2012) found parental EE to be a significant predictor of change in diagnosis. Despite differences in sample populations (inpatients vs. high‐risk) and EE subscales used as speech/language markers (criticism vs. emotional overinvolvement), the results were consistent with the literature on EE and depression across different populations, including recent‐onset psychosis patients (Rosenfarb et al. 2017), adults with depression (Hayhurst et al. 1997) and elderly patients (Hinrichsen and Pollack 1997). Of note, none of these studies employed the emerging automated approaches that use NLP for coding EE (Mirheidari et al. 2024).

Studies examining speech/language markers as predictors of psychosis onset (Agurto et al. 2020; Bearden et al. 2011; Gooding et al. 2013) used NLP more often and yielded mixed results. Manually coded negative thought disorder predicted psychosis 8 years in advance (Gooding et al. 2013) but not 1 year in advance (Bearden et al. 2011). The findings in Gooding et al. (2013) are supported by previous cross‐sectional studies, in which several sub‐measures of negative thought disorder were able to differentiate between individuals with schizophrenia and those with other mental disorders (Andreasen 1979; Ehlen et al. 2023). The discrepancy from Bearden et al. (2011) may stem from a low number of conversions to psychosis during a 1‐year follow‐up period. The use of machine learning techniques to analyse speech NLP variables, as seen in Agurto et al. (2020), has demonstrated promise, with acoustic measures significantly predicting psychosis onset. Supporting this longitudinal link, a review on acoustic patterns of schizophrenia found this type of speech marker promising (Parola et al. 2020). Furthermore, computationally derived linguistic markers that are predictive of psychosis onset appeared to be generalizable across cohorts (Bedi et al. 2015; Corcoran et al. 2018). The number of such longitudinal studies using objective markers of speech is scarce, but these preliminary findings in combination with additional cross‐sectional studies (for an overview, see Corcoran et al. 2020) underscore the potential for computational speech analysis in the prediction of psychosis onset.

Studies investigating ADHD (Allely et al. 2013; Pauli‐Pott et al. 2021; Peris and Baker 2000) focused on parental EE and vocalisation, but their predictive validity was limited for onset around 7–8 years. While the criticism subscale of EE predicted ADHD, emotional overinvolvement and positive regard did not. These findings are reflected in prior research comparing ADHD and non‐ADHD groups (Daley et al. 2003; Peris and Hinshaw 2003); however, EE has been reported to be specific to depression, rather than ADHD, and to be only slightly higher in ADHD compared with community controls of 11–12 years old (Asarnow et al. 2001). This raises concerns about the age‐related specificity of these measures. Similarly, ADHD diagnosis was not longitudinally associated with child nor maternal vocalisation by 1 year of age (Allely et al. 2013). Yet, research on speech acoustics in children with diagnosed ADHD suggests an ADHD‐related effect on such markers (Garcia‐Real et al. 2013; Nilsen et al. 2016; von Polier et al. 2025). This calls for further focused work on age‐related variable selection for predictive studies on ADHD in longitudinal settings.

When synthesising findings by speech marker type, a few patterns emerge. Acoustic markers were generally predictive of onset in MDD, appeared promising in psychosis but were understudied in both psychosis and in ADHD. However, some potential specificity of speech/language markers was observable when comparing their predictive ability across disorders. Indeed, vocal rate and frequency were predictors of psychosis but not ADHD (Agurto et al. 2020; Allely et al. 2013) and similarly spectral features (although computed differently in the two studies) were predictive of psychosis onset but not of depression. In contrast, other more subjectively rated markers such as EE showed some predictive value across disorders such as MDD and ADHD (Asarnow et al. 1993; Burkhouse et al. 2012; Peris and Baker 2000). Lastly, semantic markers of speech were exclusively studied for the prediction of psychosis, making it impossible to assess the cross‐disorder predictive ability of these markers. This is somewhat surprising given the evidence for coherence to be affected in depression as well (Palaniyappan et al. 2025).

Several studies in this review relied on small sample sizes with few outcome events, raising concerns about statistical power and model stability. For example, one of the often‐cited studies (Bedi et al. 2015) included only 34 participants, of whom 5 developed psychosis, and reported 100% classification accuracy using a convex hull classifier trained on three linguistic features. However, the classifier was evaluated on the same data it was trained on, without cross‐validation or external testing, and the low number of events (Events per variable = 1.67) suggests a high risk of overfitting despite the reported *p* < 0.05 from permutation testing. Similarly, another included study (Agurto et al. 2020) reported AUC values as high as 0.87–0.99 in a sample of 32 individuals (five CHR+), but post hoc power analysis indicated that this study had < 50% power to detect AUC = 0.75. These examples illustrate the broader challenge of underpowered predictive studies in this literature, particularly when using complex machine learning methods. Future research should prioritise larger sample sizes with adequate event counts and out‐of‐sample validation to ensure replicability.

Several limitations affect the interpretability of these findings. Many studies examined speech/language markers from parents rather than youth, limiting direct associations between youth speech and mental health outcomes (Asarnow et al. 1993; Burkhouse et al. 2012; Pauli‐Pott et al. 2021; Peris and Baker 2000). Both parental speech and the at‐risk youths' speech carry predictive signals; however, studies that collected parental EE (Asarnow et al. 1993; Burkhouse et al. 2012; Pauli‐Pott et al. 2021; Peris and Baker 2000) did not collect youth speech at the same time. Future studies should aim to collect parental speech on EEs alongside naturalistic speech from the youth to evaluate and compare their distinct, and perhaps complementary, predictive value. Additionally, nearly one‐third of studies did not report participant ethnicity, and those that did included predominantly Caucasian samples (Agurto et al. 2020; Asarnow et al. 1993; Bearden et al. 2011; Burkhouse et al. 2012; Peris and Baker 2000). Speech variables, such as EE, may differ by cultural context (Bhugra and McKenzie 2003), and these concerns should be considered even when using speech‐measurement methods based on artificial intelligence and language models (Straw and Callison‐Burch 2020). Furthermore, subjective measures, including EE, introduce potential biases, emphasizing the need for objective speech methods, such as acoustic and NLP‐based approaches (Agurto et al. 2020; Bedi et al. 2015; Corcoran et al. 2018; Peris and Baker 2000).

Developmental context—such as age and physiological stage—has a crucial impact on speech and can influence all its aspects. For example, in terms of acoustics, children and younger adolescents tend to have higher‐pitched voices, while their older counterparts typically exhibit lower pitch. The pitch gap between males and females also increases with age (Vorperian and Kent 2007). Therefore, if a model is trained solely on acoustic features from adolescents and young adults, it may not generalise well with a younger population. This is an issue that is also present in cross‐sectional studies investigating speech/language as a marker of mental disorders (Low et al. 2020). Further studies should investigate the developmental effect of speech/language markers in prospective samples from childhood to adulthood to test their predictive value across development.

Importantly, no two independent studies shared the same protocol or variables in predicting the onset of disorders. This means there are no replicable findings to date in this literature examined in this review. This lack of external validation in the literature is present in several ways, including limited out‐of‐sample testing (the only included study to do so was Corcoran et al. 2018) and significant differences in speech elicitation methods. While it is important to have models that can generalise across speech samples independently of the context, researchers must first confirm the robustness of the findings by replicating them across cohorts with shared speech task protocols. As mentioned in a previous review on speech and depression (Cummins et al. 2015), researchers should use standardised and reproducible methods to allow for comparability of findings across the literature, increase research collaboration and ensure transparency and sharing of data and code while doing so. Several other limitations and future directions are highlighted in Table 5.

**TABLE 5:** Gaps identified in the literature on speech/language markers as prospective predictors of mental disorders.

Gap domain and contributing observations	Key questions and needs
Knowledge gap: Only English and German language studies published. Studies missing for several other major mental disorders. Parental speech samples have been tested only for a subset of markers with no comparisons to children's speech.	Are the same speech/language markers predictive of psychopathology across different languages? Can we identify speech/language markers to longitudinally predict the onset of OCD, SUD, bipolar disorder or eating disorders? Can we compare the predictive ability of parent versus child speech (e.g., expressed emotion) in predicting later diagnosis?
Evidence gap: Some results are contradictory (e.g., poverty of content and psychosis). Several computational studies use the same sample. Studies need to be replicated with more complex classification models	What are the contextual factors affecting the variability in predictive performances for psychosis onset? Do acoustic and computational semantic markers generalise well to different larger cohorts? Can machine learning better predict mental disorders onset better than logistic regression or other linear methods used to date?
Empirical gap: No two independent studies shared the same protocol, variables or follow‐up duration in predicting the onset of disorders. This means there are no replicable findings to date in this literature	How does the FMSS task compare to a one‐hour long interview? How long in advance can we predict the onset of mental disorders? How does each individual marker change across lifespan?
Population gap: need find out if the results generalise to samples with different demographics or clinical presentations.	Some speech/language markers are more sensitive than others to demographic variables such as race/ethnicity, cultural background, socioeconomic status. How does this affect their predictive power? Clinical presentations can vary within a disorder (MDD with catatonic or psychotic features). Do speech/language markers generalise their predictive ability across these presentations?
Theoretical gap: psychopathology theory not applied in variable and model selection	How do acoustic markers such as spectral characterisation or vowel space inform us on the aetiology of psychosis? How do complex machine learning algorithms such as support vector machine really make their diagnostic prediction?
Methodological gap: interaction between the interviewer and interviewee is unstudied. Sentiment analysis not yet applied to analyse speech.	Are there any changes in social speech that could be captured and be predictive of later mental disorder onset. Can we quantify emotions in speech to predict later onset?
Action gap	Need for overlapping protocols of language acquisition across mental disorders to compare markers unique contribution to one mental disorder. Absence of standardised documentation for data collection, cleaning, processing workflows, model selection, parameter optimization and algorithmic choices Need for cohort studies with speech data to observe the onset of SUD, OCD, bipolar disorder and eating disorder.

Despite screening over 2000 studies, only a small number met the inclusion criteria, highlighting the scarcity of longitudinal research on speech/language markers in youth mental health. Several disorders, including OCD, EDs, SUD and BD, were not represented in any eligible studies. These gaps echo those from a prior review on speech as a cross‐sectional marker of mental disorders (Low et al. 2020), suggesting a more global need for more research on mental disorders other than psychosis, depression and ADHD and suggesting that it is too early to conclude that speech/language markers are longitudinal predictors of all the major mental disorders included in this study.

Recent advances in machine learning and NLP present promising opportunities for mental health prediction (Glaz et al. 2021); however, only three of the reviewed studies used NLP or advanced machine learning models (Agurto et al. 2020; Bedi et al. 2015; Corcoran et al. 2018), all of which focused on psychosis. Future research should expand these methods to test their predictive ability for different mental disorders in the context of speech research.

Finally, ethical issues surrounding the topic of mental disorder prediction in youth remain. Researchers have pointed to concerns of privacy and informed consent (Loch et al. 2022). Given the private nature of speech recordings, we recommend using protocols that avoid personal identifiers (e.g., those developed by the Diverse International Scientific Consortium for Research in Thought, Language and Communication in Psychosis or DISCOURSE), obtain dynamic informed consent that allows individuals to withdraw their data over time and provide options to opt out of sharing voice data with other researchers. Furthermore, the diagnosis of a mental disorder can exert a reflexive or looping effect, whereby the individual's awareness of their condition shapes and possibly alters their own self‐perception (Hacking 1995). Thus, labelling a child as at risk of developing a mental disorder could have an involuntarily negative impact on this same child's mental health. As in other studies using precision psychiatry, several considerations on communicating individual‐level risk must be incorporated in speech/language studies (Fusar‐Poli et al. 2022).

## 5 Conclusion

This systematic review highlights the potential of speech/language markers as longitudinal predictors of youth mental health changes. Despite an extensive initial search, only a handful of studies met the inclusion criteria, reflecting the scarcity of longitudinal data in this area. Despite methodological inconsistencies limiting generalizability, findings suggest that EE and FTD may predict changes in MDD, ADHD and psychosis diagnoses. The reliance on subjective speech measures underscores the need for objective approaches, such as acoustic and NLP‐based analyses. Additionally, the underrepresentation of diverse populations raises concerns about the cultural validity of speech‐based predictive models. Few studies have examined disorders beyond MDD, ADHD and psychosis, revealing a broader research gap. Future studies should prioritise diverse, large‐scale and methodologically rigorous research using standardised speech analysis techniques. Collaborative data sharing and methodological transparency will be essential for advancing speech‐based prediction models and improving early detection and intervention for youth mental health.

## Conflicts of Interest

Dr. Lena Palaniyappan reports personal fees for serving as chief editor from the Canadian Medical Association Journals, speaker/consultant fees from Janssen Canada and Otsuka Canada, SPMM Course Limited, UK, Canadian Psychiatric Association; book royalties from Oxford University Press; investigator‐initiated educational grants from Janssen Canada, Sunovion and Otsuka Canada outside the submitted work. The other authors declare no conflicts of interest.

## Data Availability

The authors have nothing to report.

## Appendix A

**Table:**

Database	Keywords
PubMed	((((((“mood disorder*”[Title/Abstract] OR “Bipolar Disorder”[MeSH Terms] OR “mdd”[Title/Abstract] OR “depressi*”[Title/Abstract] OR “bipolar*”[Title/Abstract] OR “schizo*”[Title/Abstract] OR “Schizophrenia Spectrum and Other Psychotic Disorders”[MeSH Terms] OR “psychosis”[Title/Abstract] OR “psychotic”[Title/Abstract] OR “eating disorder*”[Title/Abstract] OR “substance use disorder”[Title/Abstract] OR “addiction”[Title/Abstract] OR “obsessive compulsive disorder”[Title/Abstract] OR “ocd”[Title/Abstract] OR “attention deficit*”[Title/Abstract] OR “adhd”[Title/Abstract] OR “hyperactiv*”[Title/Abstract] OR “mental health”[Title/Abstract] OR “mental illness”[Title/Abstract] OR “psychiatr*”[Title/Abstract]) AND (“longitudinal”[Title/Abstract] OR “predict*”[Title/Abstract] OR “prospect*”[Title/Abstract] OR “Longitudinal Studies”[MeSH Terms] OR “Prospective Studies”[MeSH Terms] OR “Follow‐Up Studies”[MeSH Terms]) AND (“young”[Title/Abstract] OR “adolescent”[Title/Abstract] OR “child*”[Title/Abstract] OR “infan*”[Title/Abstract] OR “young adult”[Title/Abstract] OR “youth*”[Title/Abstract] OR “pubert*”[Title/Abstract] OR “baby”[Title/Abstract] OR “babies”[Title/Abstract]) AND (“Linguistics”[MeSH Terms] OR “Speech”[MeSH Terms] OR “Speech”[Title/Abstract] OR “linguistic*”[Title/Abstract] OR “phonetic*”[Title/Abstract] OR “semantic*”[Title/Abstract] OR “grammar”[Title/Abstract] OR “syntax”[Title/Abstract] OR “Voice Quality”[MeSH Terms] OR “Phonation”[MeSH Terms] OR (“intonation*”[Title/Abstract] AND (“Linguistics”[MeSH Terms] OR “Speech”[MeSH Terms] OR “Speech”[Title/Abstract] OR “linguistic*”[Title/Abstract])))) NOT “autis*”[Title/Abstract]) NOT “ASD”[Title/Abstract]) NOT “speech disorder”[Title/Abstract]) NOT “language disorder”[Title/Abstract]) NOT “systematic review”[Title/Abstract]
Ovid	APA PsycInfo <1806 to October 2024 Week 3> exp Phonetics/ exp speech characteristics/ vocalization/ or voice/ exp grammar/ linguistics/ or exp language/ or exp pragmatics/ or exp prosody/ or exp verbal communication/ (speech or vocalization or voice or linguistic* or prosod* or semantic* or syntax or language or phonetic* or grammar or pragmatics or voice*).ti,ab. ((speech or voice) and (pause* or pitch* or rate* or rhythm*)).ti,ab. 1 or 2 or 3 or 4 or 5 or 6 or 7 (young adult or youth or adolescen* or teen* or child*).ti,ab. (young adj3 (adult* or person* or people)).ti,ab. 9 or 10 longitudinal studies/ or prospective studies/ or followup studies/ (longitudinal or prospective or follow up).ti,ab. 12 or 13 8 and 11 and 14 affective psychosis/ or alcohol induced psychotic disorders/ or brief psychotic disorder/ or delusional disorder/ or paranoid psychosis/ or reactive psychosis/ or schizophrenia/ or substance induced psychotic disorders/ major depression/ or schizoaffective disorder/ exp “substance related and addictive disorders”/ attention deficit disorder/ anorexia nervosa/ or “avoidant/restrictive food intake disorder”/ or binge eating disorder/ or bulimia/ or feeding disorders/ or “purging (eating disorders)”/ obsessive compulsive disorder/ (mood disorder* or “Bipolar Disorder” or mdd or depressi* or bipolar* or schizo* or psycho* or “eating disorder*” or “substance use disorder” or addiction or “obsessive compulsive disorder” or ocd or “attention deficit*” or adhd or hyperactiv* or anorexia or bulimia or eating disorder* or binge eat* or alcoholic or alcoholism or substance abuse or addiction).ti,ab. 16 or 17 or 18 or 19 or 20 or 21 or 22 15 and 23 (autis* or “speech disorder” or “language disorder”).ti,ab. exp autism spectrum disorders/ exp communication disorders/ or exp language disorders/ 25 or 26 or 27 24 not 28 “systematic review”.ti,ab. 29 not 30
Google Scholar	(speech|language|voice|grammar|prosody)(adolescent|child|youth)(depression|eating disorder|ocd|psychosis|mood disorder|addiction|substance abuse|adhd)
